# Perilipin1 Expression as a Prognostic Factor in Patients with Squamous Cell Carcinoma of the Lung

**DOI:** 10.3390/diagnostics13223475

**Published:** 2023-11-19

**Authors:** Min Hye Kim, Jeong Hee Lee, Jong Sil Lee, Dong Chul Kim, Jung Wook Yang, Hyo Jung An, Ji Min Na, Wook Jae Jung, Dae Hyun Song

**Affiliations:** 1 Department of Pathology, Gyeongsang National University Hospital, Jinju 52727, Republic of Korea; joymine86@naver.com (M.H.K.);; 2 Department of Pathology, Gyeongsang National University School of Medicine, Jinju 52727, Republic of Korea; 3 Institute of Medical Science, Gyeongsang National University, Jinju 52727, Republic of Korea; 4 Department of Pathology, Gyeongsang National University Changwon Hospital, Changwon 51472, Republic of Korea; 5 Department of Pathology, Samsung Medical Center, Sungkyunkwan University School of Medicine, Seoul 06351, Republic of Korea

**Keywords:** lung cancer, squamous cell carcinoma, perilipin, prognosis, lipid metabolism

## Abstract

Perilipin (PLIN) is a major structural protein located on the surface of lipid droplets. PLIN plays an important role in human metabolism and is associated with metabolic diseases, such as obesity, diabetes, hypertension, and endocrine disorders. The dysregulation of lipid metabolism is one of the most prominent metabolic changes observed in cancers. Therefore, the PLIN protein family has recently attracted attention owing to its role in lipid metabolism and cancer. To date, no studies have addressed the association between the prognosis of lung cancer and PLIN1 expression. For the first time, we found that high PLIN1 expression was significantly correlated with worse disease-free survival (DFS) in lung squamous cell carcinoma (SCC). We examined PLIN1 expression by the immunohistochemical analysis of surgical lung SCC specimens obtained from 94 patients. We analyzed the correlation between PLIN1 expression, clinicopathological data, and patient survival, using a chi-squared test, Kaplan–Meier analysis with log-rank tests, and the multivariate Cox proportional hazards regression test. High PLIN1 expression was significantly correlated with lower DFS in the Kaplan–Meier analysis and the multivariate Cox proportional hazards regression model. High PLIN1 expression was significantly correlated with worse prognosis in lung SCC.

## 1. Introduction

Lung cancer is the most commonly diagnosed cancer and one of the leading causes of cancer-related deaths in both men and women [1]. Squamous cell carcinoma (SCC) is a type of non-small cell lung cancer and accounts for 30% of all lung cancers. SCC is associated with smoking and other risk factors, including age, second-hand smoking, and exposure to metal particles or asbestos [2].

SCC is highly aggressive, and its prognosis is poor because of the lack of targeted therapy and late detection [2]. Even patients with resectable SCC have high recurrence rates. However, few studies have investigated SCC prognosis [3]. Reliable prognostic biomarkers are required to identify high-risk patients.

Perilipin (PLIN) is a major structural protein located on the surface of lipid droplets (LD). Lipid droplets (LD) are organelles that store fat and consist of a neutral lipid core surrounded by triacylglycerides (TG), sterol esters (SE), and a phospholipid monolayer. Several proteins on the surface of LDs are involved in regulating lipid metabolism, the most well-known of which are those belonging to the PLIN protein family [4].

Perilipin1 (PLIN1), the first discovered member of the PLIN protein family, maintains the stability of LD and acts as a valve in lipid deposition. In their non-phosphorylated state, TGs are unable to undergo hydrolysis, which results in lipid deposition. Conversely, when lipase is stimulated in an energy-deficient state, PLIN1 is phosphorylated, and it induces TG lipolysis. Therefore, PLIN plays an important role in human metabolism. Therefore, it is associated with metabolic diseases, such as obesity, diabetes, hypertension, and endocrine disorders [5].

The dysregulation of lipid metabolism is one of the most prominent metabolic changes observed in cancers. Cancer cells utilize lipid metabolism to obtain energy and the signaling molecules required for proliferation, survival, invasion, metastasis, influence on the tumor microenvironment, and response to cancer therapies [6].

Here, we focused on PLIN1, an important protein involved in lipid metabolism, and investigated the correlation between PLIN1 expression and the prognosis of lung squamous cell carcinoma.

## 2. Materials and Methods

### 2.1. Patients and Clinicopathological Data

Samples from 94 patients who underwent surgery (R0) for SCC of the lungs and postoperative adjuvant therapy by National Comprehensive Cancer Network (NCCN) guidelines at Gyeongsang National University Hospital, Jinju, Korea, between January 2002 and December 2009 were evaluated by two experienced pathologists.

Stages were determined according to the eighth guidelines of the American Joint Committee on Cancer Tumor Node Metastasis (TNM). Tumor differentiation grade was determined using the fourth edition of the World Health Organization classification system. Clinical and survival data were collected from the electronic medical records of the hospital. Disease-free survival (DFS) was defined as the time from the date of surgery to the date of cancer recurrence. Disease-specific survival (DSS) was defined as the time from the date of surgery to death from SCC of the lungs.

Smoking history was classified as non-smoking (<100 lifetime cigarettes) or smoking, which included current and past smoking history. This study was approved by the Institutional Review Board of Gyeongsang National University Hospital, and the requirement for informed consent was waived (2023-06-001).

### 2.2. Tissue Microarray Construction

Specimens were obtained by surgery and fixed overnight in buffered neutral formalin (20%). The samples were embedded in paraffin blocks. Hematoxylin and eosin-stained (HE) slides prepared using the paraffin-embedded blocks were examined, and two representative tumor areas were marked on each slide. The 3 mm-sized representative cores were selected and transplanted into new recipient tissue microarray (TMA) blocks. Two representative areas were selected to differentiate between the tumor and the area located near the invasive front.

### 2.3. Immunohistochemistry

Immunohistochemical staining was performed on 4 μm thick sections from the TMA blocks. Tissues were stained with a polyclonal anti-PLIN1 antibody (ab126639, Abcam, 1:100 dilution, Cambridge, UK) using an automated immunostainer (Benchmark Ultra, Ventana Medical Systems Inc., Tucson, AZ, USA). Adipose tissue served as a positive control for the PLIN1 antibody.

### 2.4. PLIN1 Expression

Immunohistochemical staining for PLIN1 expression was performed in TMA samples with visual counting of the tumor cells. The staining results of the tumor cells for PLIN1 were defined as high or low expression. High expression was defined as higher expression than that in inflammatory cells, and low expression was defined as the same or lower expression as that in inflammatory cells. To confirm reproducibility, two independent pathologists scored all samples in a blinded manner. When there were discrepancies, the two pathologists reached a consensus score.

### 2.5. Statistical Analysis

The correlation between PLIN1 expression and the clinicopathological variables was determined using the Chi-square test. Survival probabilities for DFS and DSS were analyzed using the Cox proportional hazards regression method. DFS and DSS were assessed using the Kaplan–Meier method with the log-rank test. *p* < 0.05 was considered as statistically significant. SPSS version 25.0 (IBM Corp., Armonk, NY, USA) was used for all analyses.

## 3. Results

### 3.1. Clinicopathological Patient Data

**Figure 1:**
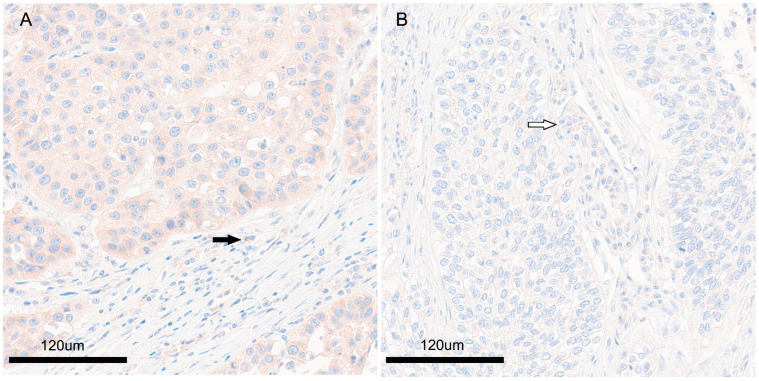
Perilipin1 expression of pulmonary squamous cell carcinoma. (**A**) High expression group, expression of perilipin1 is higher in tumor cells compared to inflammatory cells (black arrow). (**B**) Low expression group, expression of perilipin1 is lower in tumor cells compared to inflammatory cells (white arrow).

The clinicopathological data of the patients are shown in Table 1. The mean patient age was 65.5 years (range, 47 to 77). Among the 94 patients, 90 (95.7%) were men. According to the T-stage classification, the number of T1a patients was 0, T1b was 11 out of 94, accounting for 11.7%, T1c was 15 out of 94, accounting for 16.0%, T2a was 33 out of 94, accounting for 35.1%, T2b was 11 out of 94, accounting for 11.7%, T3 was 17 out of 94, accounting for 18.1%, and T4 was 7 out of 94, accounting for 7.4%. In N stage, n0 accounted for 60.6% with 57 out of 94, and n1 accounted for 36.2% with 34 out of 94. N2 was three patients, accounting for 3.2%. The histologic differentiation of SCC was well-differentiated with 14 patients, accounting for 14.9%, moderately differentiated with 58 patients, accounting for 61.7%, and poorly differentiated with 22 patients, accounting for 23.4%. Regarding the tumor, nodes, metastasis (TNM) stage: 25 patients (37.2%) had stage I, 44 (46.8%) had stage II, 14 (14.9%) had stage III, and 1 (1.1%) had stage IV. Regarding differentiation, well-differentiation was found in 14 (14.9%) cases; moderate differentiation, 58 (61.7%); and, poor differentiation, 57 (60.6%). Of these patients, 77 patients (81.9%) underwent lobectomy, 14 (14.9%) underwent pneumonectomy, and 3 (3.2%) underwent bilobectomy or sleeve lobectomy. In the immunohistochemical staining for PLIN1, 11 out of the 94 patient specimens had high expression and 83 had low expression of PLIN1. The expression of PLIN1 was exhibited in the cytoplasm of the tumor cells. The nuclear expression was inconspicuous. The pattern of expression was diffuse and homogeneous among tumor cells (Figure 1). PLIN1 was also expressed in inflammatory cells in the stroma surrounding tumor cells. PLIN1 expression was observed in the cytoplasm of inflammatory cells, and no intranuclear expression was observed. Morphologically, the inflammatory cells expressing perilipin1 were judged to be plasma cells. No expression was observed in lymphocytes, which do not have a large amount of cytoplasm.

### 3.2. Relationship between Perilipin1 Expression and Clinicopathological Characteristics of Tumor Cells

The relationship between PLIN1 expression and clinicopathological data is shown in Table 2. When cross-analyzing the association between age and PLIN1 expression, there was a pattern of more PLIN1 low expression in both those under 65 and not less than 65, with a *p* value of 0.513, which was not statistically significant. When cross-analyzing the association between gender and PLIN1 expression, males outnumbered females by 90, with a *p* value of 0.397, and no statistical significance was observed. The association of PLIN1 expression in 70 smokers and 24 non-smokers was also not statistically significant, with a *p* value of 0.552. The cross-analysis between histologic differentiation and PLIN1 expression was also not statistically significant, with a *p* value of 0.552. The correlation between tumor stage < 2 and ≥2 and PLIN1 expression was also not statistically significant with a *p* value of 0.455. The correlation between lymph node metastasis and PLIN1 expression was also not significant with a *p* value of 0.273, and the association between distant metastasis and PLIN1 expression was not statistically significant with a *p* value of 0.714. The association between TNM stage and PLIN1 expression was also not significant with a *p* value of 0.467. High PLIN1 expression did not show any significant correlation with clinicopathological characteristics.

### 3.3. Perilipin 1 Expression and Survival Analysis

**Figure 2:**
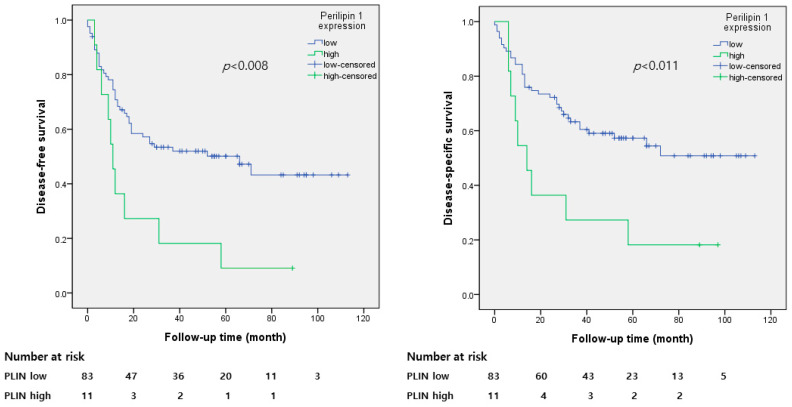
Kaplan–Meier survival analysis based on perilipin 1 expression in patients with pulmonary squamous cell carcinoma. The group with high Perilipin1 expression had significantly lower disease-free survival (*p* < 0.008) and disease-specific survival (*p* < 0.011).

The mean follow-up period was 113 months. In total, 55.3% of the patients (*n* = 52) experienced recurrence, and 47.9% (*n* = 45) died. The median DFS was 35.7 months and the mean DSS time was 42.6 months. The Kaplan–Meier analysis is shown in Figure 2. DFS and DSS were significantly lower in the group with high PLIN1 expression (*p* < 0.008 and *p* < 0.011, respectively).

Univariate analysis of Cox proportional hazards regression test showed that age ≥ 65 years was associated with a hazard of 1.386 times (*p* = 0.271, 95% CI 0.775–2.479) higher DFS and 1.158 times (*p* = 0.638, 95% CI 0.628–2.135) higher DSS than age < 65 years, but the *p* values were not statistically significant. Gender was not statistically significant for DFS with a hazard ratio of 0.802 (*p* = 0.76, 95% CI 0.195–3.298) and DSS with a hazard ratio of 0.388 (*p* = 0.35, 95% CI 0.053–2.823). The DFS hazard ratio according to T stage < 2 and ≥2 was 1.205 (*p* = 0.562, 95% CI 0.642–2.261), and the DSS was not statistically significant with a hazard ratio of 1.512 (*p* = 0.25, 95% CI 0.748–3.057). At N stage, the DFS hazard ratio for N0 and N1 or higher was 1.033 (*p* = 0.91, 95% CI 0.590–1.807), and the DSS was not statistically significant with a hazard ratio of 1.12 (*p* = 0.711, 95% CI 0.616–2.034). The DFS hazard ratio for smoking status was 0.666 (*p* = 0.179, 95% CI 0.369–1.205) and DSS was not statistically significant with a hazard ratio of 0.693 (*p* = 0.257, 95% CI 0.367–1.307). The DFS hazard ratio according to SCC differentiation was 2.263 (*p* = 0.006, 95% CI 1.267–4.039) and DSS was statistically significant with a hazard ratio of 2.188 (*p* = 0.017, 95% CI 1.183–4.045). The DFS hazard ratio according to PLIN1 expression was 2.454 (*p* = 0.011, 95% CI 1.225–4.919) and DSS was statistically significant with a hazard ratio of 2.503 (*p* = 0.014, 95% CI 1.200–5.221). Multivariate analysis was performed with SCC differentiation and PLIN1 expression, variables that were statistically significant in univariate analysis, and the DFS hazard ratio for SCC differentiation was 2.098 (*p* = 0.013, 95% CI 1.167–3.774) and DSS was statistically significant with a hazard ratio of 1.952 (*p* = 0.037, 95% CI 1.040–3.664). According to the degree of PLIN1 expression, the DFS hazard ratio was 2.178 (*p* = 0.03, 95% CI 1.078–4.399) and DSS was 2.116 (*p* = 0.051, 95% CI 0.996–4.497), which was not statistically significant.

The results of the multivariate Cox proportional hazards regression analysis are presented in Table 3. It demonstrated that high PLIN1 expression was independently associated with worse DFS (hazard ratio, 2.178; 95% confidence interval, 1.078 to 4.399; *p* = 0.03). Differentiation of SCC was an independent factor for worse DFS and DSS (hazard ratio, 2.09; 95% confidence interval, 1.167 to 3.774; *p* = 0.013and hazard ratio, 1.952; 95% confidence interval, 1.040 to 3.664; *p* = 0.037, respectively).

The clinicopathological data of the 11 PLIN1 high-expression patients are summarized in Table 4. The median age of the 11 patients was 68 years, with a mean DFS of 22.64 months. The mean DSS was 31.18 months. Two of the eleven patients had no smoking history, and the histologic differentiation was well differentiated in three, moderately differentiated in four, and poorly differentiated in four. Chemotherapy was performed in two patients.

## 4. Discussion

Characteristics of tumor cells include frequent proliferation and infiltration. The rapid proliferation of tumor cells requires increased energy. Thus, their metabolism may differ from that of normal cells [5].

In the 1920s, Warburg observed that tumor cells produced energy without undergoing the citric acid cycle or oxidative phosphorylation in the mitochondria of normal cells but converted glucose to lactic acid through the glycolytic pathway, called aerobic glycolysis or the Warburg effect. Even in the presence of sufficient oxygen, glucose uptake is increased, and lactic acid is produced. This has been the subject of many cancer-related studies [7].

In the 2000s, the relationship between lipid metabolism and cancer development was actively investigated. Many studies revealed that lipid metabolism regulates the growth, survival, and metastasis of cancer cells [8,9]. Among these, the PLIN protein family has recently attracted attention as a substance involved in lipid metabolism and cancer. In this study, we examined the association of PLIN1 with recurrence and patient survival, with the hypothesis that PLIN would increase SCC metabolism of the lung. PLIN has previously been studied for its association with patient survival in several organ cancers.

The correlation between PLIN expression and disease prognosis has been reported in kidney, breast, uterine cervix, and lung cancers. In renal cell carcinoma, PLIN2 expression was correlated with a favorable prognosis, but the high expression of PLIN3 is correlated with a poor prognosis. In breast cancer, the high expression of PLIN1 correlated with a good prognosis, but PLIN2 and PLIN4 expression correlated with a poor prognosis. In cervical cancer, high PLIN expression indicates poor prognosis. In lung cancer, the high expression of PLIN2 indicates poor prognosis [9,10].

The present study demonstrated the prognostic significance of PLIN1 expression in lung SCC. High PLIN1 expression was significantly correlated with lower DFS in the Kaplan-Meier analysis (*p* < 0.008) and multivariate Cox proportional hazards regression model (hazard ratio, 2.178; 95% confidence interval, 1.078 to 4.399; *p* = 0.03). DSS was also lower in patients with high PLIN1 expression in the Kaplan–Meier analysis, but the multivariate Cox proportional hazards model showed no significant correlation in statistics.

To date, no studies have reported on the prognosis of lung cancer and PLIN1 expression. In this study, for the first time, we found that high PLIN1 expression was significantly correlated with worse DFS in pulmonary SCC. High and low levels were defined in comparison to the surrounding inflammatory cells as high expression when the expression was similar or more and low expression when it was lower than that of the surrounding inflammatory cells. This also indicates that the expression of PLIN1 is affected by the tumor microenvironment. To the best of our knowledge, there are no other studies on the association between tumor-associated inflammation and PLIN1 in pulmonary SCC. A study by Wagner et al. suggested that tumor-associated adipose cells around breast cancer cells have decreased PLIN1 and induce tumor-associated inflammation [11]. Kitahara et al. suggested that there is an association between prognostic nutritional index (PNI) and tumor-infiltrating lymphocytes (TIL) in lung SCC [12]. Additionally, it has been suggested that PNIs and TILs are associated with local immunity and systemic immune response, affecting patient prognosis. The study of Kitahara et al. is similar to the present study, in that it suggests a link between nutritional metabolism and prognosis. However, the statistically significant correlation between PLIN1 expression in SCC cells compared with immune cells and patient prognosis described in this study requires further systematic laboratory studies to discover the mechanisms of biomolecular levels.

In carcinoma, which accounts for the majority of malignant tumors in humans and originates from the epithelial cells of major organs, there are not many studies related to PLIN, but since lipids are a major component of cell membranes and an important energy source, research on PLIN and lipid metabolism mechanisms has been ongoing on adipocytes. A study by Fruhbeck et al. suggested that PLIN is present on the surface of intracellular lipid droplets and acts as an important regulator of lipolytic mechanisms [13]. Sztalryd et al. showed that small lipid droplets are present in human cells, which are mainly associated with PLIN2 and PLIN3 [14]. Cells specialized for fat storage express PLIN1, PLIN4, and PLIN5, and they suggest that PLIN1 and PLIN5 are involved in lipid phosphorylation. The authors elaborated on the intracellular role of PLIN1: in the stabilized state of adipocytes, PLIN1 is attached to the side of fat droplets along with ABHD5 and prevents the interaction of the alpha beta hydrolase domain (ABHD) with adipose triglyceride lipase (ATGL). This keeps lipolysis low. Under lipolytically stimulated conditions, PLIN1, ABHD, and ATGL are phosphorylated, which promotes lipolysis, leading to fatty acid release. This mechanism has also been linked to an insulin-mediated mechanism. In a study comparing liposarcoma and non-lipomatous sarcoma, PLIN2 was mostly positive in both groups, but PLIN1 was positive in more than two-thirds of liposarcomas but negative in non-lipomatous sarcomas, showing a statistically significant difference (*p* < 0.001), and its usefulness as a marker of adipocytic differentiation was reported by Westhoff et al. [15].These disease-specific features of PLIN1, as well as its well-understood mechanism of action, are the reasons that PLIN1 was chosen for the present study.

Obesity its association with PLIN is also an important topic. Smith et al. reported that a knockout mouse model of PLIN1 was resistant to obesity on a high-fat diet compared to wild-type mice, and increased glucose intolerance and insulin resistance was confirmed [16]. Interestingly, PLIN1 overexpression was found to be protective against obesity, adipocyte hypertrophy, and glucose intolerance in a mouse model. They also suggested that PLIN1 is involved in the insulin mechanism of action, which plays an important role in sugar metabolism. Yang et al. showed that a decrease in O-linked β-N-acetylglucosamine transferase (OGT) decreases PLIN1 phosphorylation, and that OGT overexpression prevents lipolysis and causes diet-induced obesity [17]. These findings suggest that PLIN1 is closely related to human obesity and may play an important role in the treatment of obesity.

PLIN1 has also been studied in coronary artery disease and nonalcoholic fatty liver disease, which are major diseases associated with obesity. Eleftheriadis et al. measured serum PLIN1 in hemodialysis patients with frequent dyslipidemia, malnutrition, inflammation and atherosclerosis, and found an association between increased high-density lipoprotein cholesterol and reduced incidence of coronary artery disease [18]. The lipid metabolism association of PLIN1 also has great potential for use in the diagnosis and treatment of nonalcoholic fatty liver disease [19,20].

PLIN1 has been suggested as a useful diagnostic marker for lipodystrophy, a disease characterized by an inability to produce or maintain healthy adipose tissue in the body [21].

In the diagnosis of sarcoma, a malignant tumor of mesenchymal origin, adipocytic differentiation is an important criterion for diagnosis. Straub et al. proposed the use of PLIN1 in the diagnostic classification of soft tissue sarcomas [22]. In their study, immunohistochemical staining was performed on 478 human-derived soft tissue sarcomas and 60 human-derived normal tissues. All 24 well-differentiated liposarcomas were positive for PLIN1, 97.3% of 30 myxoid liposarcomas were positive for PLIN1, 72.7% of 13 pleomorphic liposarcomas were positive for PLIN1, and all 15 lipomas or angiolipomas were positive for PLIN1. In contrast, 65 myxofibrosarcoma and fibromatosis, 70 leiomyosarcoma and rhabdomyosarcoma, 38 malignant peripheral nerve sheath tumor neurofibroma, and schwannoma, 40 synovial sarcoma, and 63 undifferentiated pleomorphic sarcoma were all PLIN-negative. The results showed the potential of PLIN1 as a useful diagnostic marker in the pathological reading of soft tissue sarcomas. However, in 48 dedifferentiated liposarcomas, PLIN1 was positive only in the differentiated component. In dedifferentiated sarcomas, pleomorphic liposarcomas, and other high-grade sarcomas, PLIN2 was more distinctly positive, suggesting its potential as a therapeutic target.

The characterization of PLIN1 expression in cells with adipocytic differentiation has been applied in the medical field, including the quantitative analysis of intramedullary adipocytes in bone marrow tissue diagnosis. According to a study by Widjaja et al., intramedullary adipocytes are not thought to be nonfunctional tissues that fill bone marrow, but rather tissues involved in various metabolic processes [23]. There are many attempts to quantify bone marrow adipocytes (BMA), but there are limitations in quantitative analysis due to the manual method guided by a simple microscopic counting, so they utilized the adipocyte specific feature of PLIN1 for quantitative analysis after immunostaining. The samples were the femur and tibia of 24-week-old male rats, and they reported good results with statistical significance.

Sanchez-Romero et al. performed PLIN1 immunohistochemical staining in ameloblastoma and ameloblastic carcinoma, tumors that originate from tooth germ cells [24]. Twelve human-derived tooth germ tissues (TG), twenty-seven conventional ameloblastomas (AM), and eight ameloblastic carcinomas (AC) were enrolled. A total of 8.3% of TG, 48.2% of AM, and 87.5% of AC were positive for PLIN1. This result suggests the potential of PLIN1 as a prognostic predictor, as AC is the disease with the worst prognosis.

PLIN1 is the most well-characterized member of the PLIN family. In the present study, we found that the PLIN1 high-expression SCC group had a statistically significantly worse DFS than the low-expression group. This is the first report by our group in the literature review. There have been a few studies on the association between the consumption of meat, the main source of lipids in the human body, and lung SCC. Deneo-Pellegrini et al. reported that meat consumption was a strong risk factor for lung SCC in a case–control study of Uruguayan men [25]. They enrolled 300 patients with lung SCC as a case group and 600 patients with non-neoplastic disease as a control group, and conducted interviews and questionnaires to obtain the results. Although it is limited by the fact that it is not a cohort study, the finding that meat consumption is associated with the development of lung SCC shares some hypotheses with our study, in that it suggests a lipid metabolism is related to lung SCC. Lam et al. also reported that red meat, processed meat, and meat-mutagens were independently associated with lung cancer incidence [26]. This large-scale interview survey study in the United States included 1903 lung cancer patients and 2073 non-cancerous individuals. Red and processed meat consumption was found to be a statistically significant risk factor for lung cancer even in patients who had never smoked. Based on these findings, lipids and lipid metabolism are likely to be important survival mechanisms in lung cancer cells. Our findings that PLIN1 expressed in the cytoplasm of lung SCC affects patient DFS are in line with the above studies.

## 5. Conclusions

High PLIN1 expression is significantly correlated with worse prognosis in lung SCC.

## Figures and Tables

**Table 1:** Clinicopathological data of the patients (*n* = 94).

Clinicopathological Data of the Patients	Number (%) (*n* = 94)	
Mean age, yr	65.6	
Male gender	90	(95.7)
Surgery		
Lobectomy	77	(81.9)
Bilobectomy or sleeve	3	(3.2)
Pneumonectomy	14	(14.9)
T stage		
T1a	0	(0)
T1b	11	(11.7)
T1c	15	(16.0)
T2a	33	(35.1)
T2b	11	(11.7)
T3	17	(18.1)
T4	7	(7.4)
N stage		
n0	57	(60.6)
n1	34	(36.2)
n2	3	(3.2)
Differentiation		
W/D	14	(14.9)
M/D	58	(61.7)
P/D	22	(23.4)
Smoker, ex-smoker	70	(74.5)
Lymph node metastasis		
n0	57	(60.6)
n1	34	(36.2)
n2	3	(3.2)
Perilipin1		
Low	83	(88.3)
High	11	(11.7)

yr, year; sleeve, sleeve lobectomy; W/D, well-differentiated; M/D, moderately differentiated; P/D, poorly differentiated.

**Table 2:** Correlation of perilipin1 expression with clinicopathological data in patients with squamous cell carcinoma of the lung.

Factor		Perilipin1		*p*-Value
	Subgroup	Low	High	
Age	<65 Years	31	3	0.513
	≥65 years	52	8	
Sex	Male	80	10	0.397
	Female	3	1	
Smoking	Non-smoker	22	2	0.552
	Smoker	61	9	
Surgery	Lobectomy	68	9	0.993
	Bilobectomy or Pneumonectomy	15	2	
Histologic differentiation	W/D	11	3	0.176
	M/D	54	4	
	P/D	18	4	
Tumor stage	<2	24	2	0.455
	≥2	59	9	
Lymph node metastasis	Absent	52	5	0.273
	Present	31	6	
Distant metastasis	Absent	82	11	0.714
	Present	1	0	
TNM	I	32	3	0.467
	II, III, IV	51	8	

W/D, well-differentiated; M/D, moderately differentiated; P/D, poorly differentiated; TNM, tumor-nodes-metastasis stage.

**Table 3:** Cox proportional hazards regression test of disease-free and disease-specific survival for patients with pulmonary squamous cell carcinoma.

**Variables**	Univariate Analysis				Multivariate Analysis			
	DFS		DSS		DFS		DSS	
	HR	*p*-Value	HR	*p*-Value	HR	*p*-Value	HR	*p*-Value
	(95% CI)		(95% CI)		(95% CI)		(95% CI)	
Age, yr	1.386	0.271	1.158	0.638				
(<65 vs. ≥65)	(0.775–2.479)		(0.628–2.135)					
Sex	0.802	0.760	0.388	0.35				
(male vs. female)	(0.195–3.298)		(0.053–2.823)					
T stage	1.205	0.562	1.512	0.25				
(<2 vs. ≥2)	(0.642–2.261)		(0.748–3.057)					
N stage	1.033	0.910	1.12	0.711				
(N0 vs. N1,2,3)	(0.590–1.807)		(0.616–2.034)					
Smoking	0.666	0.179	0.693	0.257				
(non vs. current, ex-smoker)	(0.369–1.205)		(0.367–1.307)					
Surgery	1.509	0.216	1.432	0.319				
(lobectomy vs. bilobectomy, pneumonectomy)	(0.786–2.895)		(0.707–2.903)					
SCC differentiation	2.263	0.006	2.188	0.017	2.098	0.013	1.952	0.037
(W/D, M/D vs. P/D)	(1.267–4.039)		(1.183–4.045)		(1.167–3.774)		(1.040–3.664)	
Perilipin1	2.454	0.011	2.503	0.014	2.178	0.03	2.116	0.051
(low vs. high)	(1.225–4.919)		(1.200–5.221)		(1.078–4.399)		(0.996–4.497)	

DFS, disease-free survival; DSS, disease-specific survival; HR, hazard ratio; yr, year; SCC, squamous cell carcinoma.

**Table 4:** Clinicopathological data of 11 patients with perilipin 1 high expression.

No. of Patient	Age	Sex	Smoking	Surgery	TNM Stage_8th	Pathologic Differentiation	Period of DFS (Month)	Recur	Period of DSS (Month)	Death for Lung Cancer	Chemo Tx.
1	70	male	current, ex-smoker	lobectomy	IA3	P/D	3	yes	6	yes	yes
2	68	female	current, ex-smoker	lobectomy	IIB	P/D	11	yes	97	no	NI
3	72	male	current, ex-smoker	lobectomy	IIB	M/D	4	yes	7	yes	no
4	71	male	current, ex-smoker	pneumonectomy	IIIA	M/D	9	yes	9	yes	no
5	67	male	Nonsmoker	bilobectomy or sleeve op	IIB	W/D	6	yes	6	yes	yes
6	68	male	current, ex-smoker	lobectomy	IIB	P/D	12	yes	14	yes	no
7	63	male	current, ex-smoker	lobectomy	IIA	W/D	58	yes	58	yes	no
8	62	male	current, ex-smoker	lobectomy	IIB	M/D	16	yes	16	yes	no
9	69	male	current, ex-smoker	lobectomy	IB	M/D	89	no	89	no	no
10	67	male	current, ex-smoker	lobectomy	IB	P/D	31	yes	31	yes	no
11	60	male	Nonsmoker	lobectomy	IIB	W/D	10	yes	10	yes	yes

No., number; DFS, disease-free survival; DSS, disease-specific survival; Tx., therapy; op, operation; W/D, well differentiated; M/D, moderately differentiated; P/D, poorly differentiated; NI, not identified.

## Data Availability

The datasets used and/or analyzed during the current study are available from the corresponding author upon reasonable request.
